# Distribution of Staphylococcal Cassette Chromosome mec Types and Correlation with Comorbidity and Infection Type in Patients with MRSA Bacteremia

**DOI:** 10.1371/journal.pone.0009489

**Published:** 2010-03-05

**Authors:** Jiun-Ling Wang, Jann-Tay Wang, Shey-Ying Chen, Yee-Chun Chen, Shan-Chwen Chang

**Affiliations:** 1 Department of Internal Medicine, National Taiwan University Hospital, National Taiwan University, Taipei, Taiwan; 2 Department of Internal Medicine, E-Da Hospital/I-Shou University, Kaohsiung County, Taiwan; 3 Department of Emergency Medicine, National Taiwan University Hospital, National Taiwan University, Taipei, Taiwan; National Institute of Allergy and Infectious Diseases, National Institutes of Health, United States of America

## Abstract

**Background:**

Molecular epidemiological definitions that are based on staphylococcal cassette chromosome *mec* (SCC*mec*) typing and phylogenetic analysis of methicillin-resistant Staphylococcus aureus (MRSA) isolates are considered a reliable way to distinguish between healthcare-associated MRSA (HA-MRSA) and community-associated MRSA (CA-MRSA). However, there is little information regarding the clinical features and outcomes of bacteremia patients with MRSA carrying different SCC*mec* types.

**Methods:**

From January 1 through December 31, 2006, we recorded the demographic data and outcomes of 159 consecutive adult MRSA bacteremia patients from whom isolates for SCC*mec* analysis were collected. All participants were patients at a tertiary care center in Taiwan.

**Principal Findings:**

The following SCC*mec* types were identified in MRSA isolates: 30 SCC*mec* II (18.9%), 87 SCC*mec* III (54.7%), 22 SCC*mec* IV (13.8%), and 20 SCC*mec* V (12.6%). The time from admission to the first MRSA-positive blood culture for patients infected with isolates with the SCC*mec* III element (mean/median, 50.7/26 days) was significantly longer than for patients infected with isolates carrying SCC*mec* IV or V (mean/median, 6.7/3 days for SCC*mec* IV; 11.1/10.5 days for SCC*mec* V) (*P*<0.05). In univariate analysis, community onset, soft tissue infection, and deep-seated infection were predictors for SCC*mec* IV/V. In multivariate analysis, length of stay before index culture, diabetes mellitus, and being bedridden were independent risk factors associated with SCC*mec* II/III.

**Conclusions:**

These findings are in agreement with previous studies of the genetic characteristics of CA-MRSA. MRSA bacteremia with SCC*mec* II/III isolates occurred more among patients with serious comorbidities and prolonged hospitalization. Community onset, skin and soft tissue infection, and deep-seated infection best predicted SCC*mec* IV/V MRSA bacteremia.

## Introduction

Community-associated methicillin-resistant *Staphylococcus aureus* (CA-MRSA) has been isolated mainly from skin or soft tissue infections [1], [2], although severe invasive infections caused by CA-MRSA strains, such as pyomyositis, osteomyelitis, necrotizing fasciitis, severe pneumonia, and sepsis, have also been reported [3]–[7]. CA-MRSA strains share a common pulsed field gel electrophoresis (PFGE) pattern, show greater susceptibility to most non-β-lactam antibiotics, and carry staphylococcal cassette chromosome *mec* (SCC*mec*) type IV or V elements; these characteristics are not typical of healthcare-associated MRSA (HA-MRSA) strains [1].

In recent years, CA-MRSA has emerged as an important cause of healthcare-associated and nosocomial bacteremia in many countries [8]–[10]. In contrast to the US and Europe, the major CA-MRSA strains in Taiwan are ST59, SCC*mec* IV or ST59, SCC*mec* V_T_; the major clone in nosocomial MRSA infections in Taiwan is SCC*mec* III: ST239, followed by SCC*mec* II: ST 5 [11]–[16]. Molecular epidemiological definitions, based on staphylococcal cassette chromosome *mec* (SCC*mec*) typing and phylogenetic analysis of MRSA isolates, are considered the most reliable way to distinguish between HA-MRSA and CA-MRSA [17]. In a study of MRSA bacteremia by Seybold et al., CA-MRSA strains (as defined by PFGE) were associated with injection drug use and with skin and soft tissue infection [8]. In their study of CA-MRSA strains defined by antibiogram phenotype, Popovich et al. concluded that demographic data and risk factors could not reliably distinguish patients infected with CA-MRSA strains from those infected with HA-MRSA strains [9].

SCC*mec* II and III, which are larger elements, may not be suited to CA-MRSA strains; these elements show a different distribution of antibiotic resistance genes and toxin genes compared to SCC*mec* IV and V [17]. In this study, we compared the demographic characteristics, clinical features, and outcomes of patients with MRSA isolates with different SCC*mec* types.

## Materials and Methods

### Patient Selection

This study was conducted according to the principles expressed in the Declaration of Helsinki and was approved by the Institutional Review Board (No. 200705068R) of National Taiwan University Hospital (NTUH), Taipei, Taiwan. The Institutional Review Board waived the need for informed consent from participants because the study involved very minimal risk to the subjects, did not include intentional deception, and did not involve sensitive populations or topics; this waiver does not adversely affect the rights and welfare of the subjects.

NTUH is a university hospital with 2500 beds that provides primary and tertiary care in northern Taiwan. From January 1 through December 31, 2006, all patients age >16 years with MRSA bacteremia admitted to NTUH were identified from information in a laboratory database.

### Data Collection

All patients were evaluated using a structured form. The clinical course of the infection and the infection foci were evaluated and recorded based on information supplied by primary care physicians and medical records. Diagnosis of the infection focus was based on clinical, bacteriological, and radiological results. The infection was considered “deep-seated” if any of the following were present: infective endocarditis, mycotic aneurysm, osteomyelitis, septic arthritis, pyomyositis, necrotizing pneumonia/empyema, or abscess formation in any deep organ, such as the liver or kidney. Modified Duke criteria were used to evaluate infective endocarditis [18]. Catheter-related bacteremia was defined by a semi-quantitative culture of the vascular catheter tip that yielded more than 15 MRSA colonies in the absence of other sources of bacteremia [19]. The other sites of infection at the onset of bacteremia were defined according to the US Centers for Disease Control and Prevention criteria [20]. If no infection focus could be identified, the bacteremia was classified as primary bacteremia.

The following data were recorded for each patient: age, sex, underlying illness, hospitalization history or outpatient department involvement within the previous year, existence of a percutaneous device catheter, time from admission until a MRSA-positive culture, initial laboratory findings, and outcome.

“Health care–associated” was defined using previously published definitions [8], [9], which include hospital-onset infection or the presence of any of the following HA-MRSA risk factors within the year prior to the index culture: (1) residence in a longterm care facility, (2) prior admission to an acute care facility (3) use of central intravenous catheters or longterm venous access devices, (4) use of urinary catheters, (5) use of other longterm percutaneous devices (6) prior surgical procedures, and/or (7) need for any form of dialysis [8], [9].

### Microbiological Laboratory Procedures

Identification of *S.aureus* was based on colony morphology, Gram staining, a positive catalase reaction, slide agglutination test results (bioMérieux, Marcy l'Etoile, France), and/or results obtained with the Phoenix system (Becton Dickinson, Sparks, MD, USA). Antibiotic susceptibility testing for *S. aureus* in this study included oxacillin, vancomycin, minocycline, levofloxacin, ciprofloxacin, tetracycline, trimethoprim/sulfamethoxazole, gentamicin, clindamycin, and rifampin and was performed according to standard microbiological methods [21]. Resistance to oxacillin was confirmed by PCR for the *mec*A gene. The presence of the PVL gene lukF-lukS was determined by PCR using a primer that was described previously [22], and the presence of 10 staphylococcal enterotoxin virulence genes (sea, seb, sec, sed, see, seg, seh, sei, sej, and tst) was also determined by PCR using the protocol of Jarraud et al. [23]. The presence of the SCC*mec* elements (I–V) and the *mec*A gene was determined by methods described previously [14], [24]–[26]. Analysis of the polymorphic X-region of the protein A gene (spa) and multilocus sequence typing (MLST) for the major pulso type was performed as described previously [27]–[28]. Analysis of PFGE patterns was performed using GelCompar software (Applied Maths, Austin, TX, USA). We performed spa typing and PFGE typing of all isolates, but only performed MLST on major pulsotypes in different SCC*mec*types.

### Statistical Analysis

Mean values and standard deviations were calculated for continuous variables. Percentages were used for categorical variables. For univariate analysis, SCC*mec* types were compared using the chi-square test or Fisher's exact test as indicated for categorical variables and the analysis of variance (ANOVA) test with least-significant-difference post-hoc tests for continuous variables. Due to their similar biological features, we considered SCC*mec* II and III to be one group and SCC*mec* IV and SCC*mec* V to be another group. The associations between potential risk factors and SCC*mec* II/III or SCC*mec* IV/V in patients with MRSA bacteremia were also investigated using univariate and multivariate logistical regression. Crude and adjusted odds ratios (ORs) and the corresponding 95% confidence intervals (CIs) were calculated. Cumulative survival after the day of the first MRSA-positive blood culture was calculated using the Kaplan-Meier method. Differences in cumulative survival for patients with different SCC*mec* types were tested with the log-rank test. The effect of infection with CA-MRSA on outcome was evaluated using a multivariate Cox proportional hazards regression model adjusted for age, sex, and underlying comorbidities. The data were analyzed using SPSS software for Windows (Release 15.0; SPSS, Chicago, IL).

## Results

### Patients, Risk Factors, and Clinical Features

**Figure 1:**
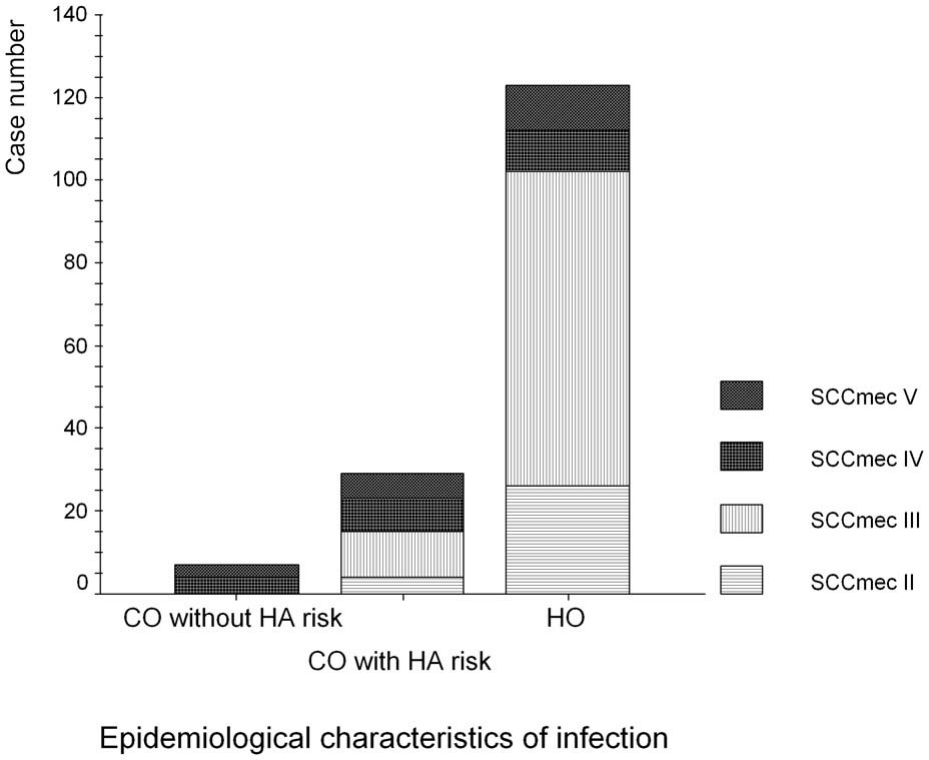
The distribution of SCCmec: community-onset (CO) or hospital-onset (HO) and healthcare-associated (HA) factor.

During the study period, there were 159 consecutive adult patients with MRSA bacteremia from whom isolates were collected for microbiological analysis (101 men, 63.5%; 58 women, 36.5%). Twelve patients from whom isolates were not collected were excluded from the study. There were no differences between the study group and the excluded group in terms of sex, age, and ward distribution. The mean age of the 159 adult patients was 67.3±16.5 years. The MRSA SCC*mec* types were as follows: 30 SCC*mec* II (18.9%), 87 SCC*mec* III (54.7%), 22 SCC*mec* IV (13.8%), and 20 SCC*mec* V (12.6%). All SCC*mec* V isolates belonged to SCC*mec* type V_T_ [13]. Seven patients matched the criteria for CA-MRSA bacteremia (4.4%), including 4 (57.1%) MRSA isolates with SCC*mec* IV and 3 (42.9%) with SCC*mec* V. In the remaining 152 (95.6%) patients with HA-MRSA bacteremia, 29 (18.2%) patients had community-onset infections and 123 (77.4%) had hospital-onset infections. For 29 patients with community-onset HA-MRSA bacteremia, the SCC*mec* type distribution was as follows: 4 (13.8%) SCC*mec* II, 11 (37.9%) SCC*mec* III, 8 (27.6%) SCC*mec* IV, and 6 (20.7%) SCC*mec* V. In 123 patients with hospital-onset HA-MRSA bacteremia, the distribution of SCC*mec* types was 26 (21.1%) SCC*mec* II, 76 (61.3%) SCC*mec* III, 10 (8.1%) SCC*mec* IV, and 11 (8.9%) SCC*mec* V. The distribution of each SCC*mec* type in a heathcare setting (either community-onset or hospital-onset, with or without healthcare associated factor) is shown in Figure 1.

### MRSA Bacteremia Patient Characteristics Associated with Specific MRSA SCC*mec* Types

Table 1 summarizes the demographic data and comorbidities of all patients. The mean age of patients with MRSA SCC*mec* V (56.9 years) was significantly lower than that of patients with MRSA SCC*mec* II (69.9) and MRSA SCC*mec* III (69.0) (*P*<0.05). MRSA carrying SCC*mec* II/III was more likely to occur in the ICU, and MRSA carrying SCC*mec* IV/V was more likely to occur in the community (*P*<0.05). The time from admission to the first MRSA-positive blood culture for patients infected with isolates with the SCC*mec* III element (mean/median, 50.7/26 days) was significantly longer than for patients infected with isolates carrying SCC*mec* IV or V (mean/median, 6.7/3 days for SCC*mec* IV; 11.1/10.5 days for SCC*mec* V) (*P*<0.05). Compared to patients with SCC*mec* V isolates, MRSA bacteremia patients with SCC*mec* III isolates were more likely to be bedridden, but their infections were less likely to be associated with underlying disease (*P*<0.05). Patients with SCC*mec* III isolates were also more likely to have diabetes compared to those with MRSA SCC*mec* IV (*P*<0.05). Compared to patients with SCC*mec* IV isolates, those with MRSA carrying SCC*mec* II were more likely to have had surgery within the previous three months (*P*<0.05)

**Table 1:** Comparison of demographic data and comorbidities of MRSA bacteremia patients with different SCCmec types.

Characteristic, n (%)	SCC*mec* II n = 30	SCC*mec* III n = 87	SCC*mec* IV n = 22	SCC*mec* V n = 20	Total n = 159	*P*
Sex, male	20 (66.7)	56 (64.4)	12 (54.5)	13 (65.0)	101 (63.5)	0.816
Age, mean years ± SD	69.9±14.9	69.0±15.9	66.6±15.6	57.0±19.4	67.3±16.5	0.02
Community onset	4 (13.3)	11 (12.6)	12 (54.5)	9 (45.0)	36 (22.6)	<0.001
ICU onset	15 (50)	40 (46)	2 (9.1)	0 (0)	57 (35.8)	<0.001
Length of hospital say before index culture mean days ± SD, median (range)	35.9±33.1 28 (0–157)	50.7±71.3 26 (0–360)	6.7±9.0 3 (0–33)	11.1±12.2 10.5 (0–39)	36.8±57.6 19.0 (0–360)	0.001
Comorbid condition						
No underlying disease	0 (0)	0 (0)	1 (4.8)	2 (10)	3 (1.9)	0.017
Diabetes mellitus	7 (23.3)	35 (40.2)	2 (9.1)	6 (30.0)	50 (31.4)	0.027
Cancer	10 (33.3)	25 (28.7)	5 (22.7)	11 (55)	51 (32.1)	0.106
Liver cirrhosis	2 (6.7)	12 (13.8)	1 (4.5)	4 (20)	19 (11.9)	0.375
End-stage renal disease	3 (10)	16 (18.4)	4 (18.2)	1 (5)	24 (15.1)	0.409
Cerebrovascular disease	5 (16.7)	24 (27.6)	3 (13.6)	2 (10)	34 (21.4)	0.242
Charlson comorbidity score ≥3	17 (56.7)	55 (63.2)	10 (45.5)	11 (55)	93 (58.5)	0.490
Bed-ridden status	7 (23.3)	28 (32.2)	3 (13.6)	1 (0.5)	39 (24.5)	0.035
Recent surgery	10 (33.3)	16 (18.4)	0 (0)	2 (10.0)	28 (17.6)	0.009

Table 2 shows the clinical characteristics and outcomes of bacteremia patients infected with MRSA with different SCC*mec* types. In terms of infection sites, MRSA with SCC*mec* IV was more often associated with superficial skin and soft tissue infections than MRSA with SCC*mec* II (*P* = 0.015). In addition, patients with MRSA carrying SCC*mec* IV were significantly more likely to have non-prosthetic septic arthritis/osteomyelitis (*P*<0.001) and deep-seated infections not related to surgery or prosthesis (*P* = 0.008) compared to MRSA bacteremia patients with SCC*mec* II and SCC*mec* III isolates.

**Table 2:** Comparison of clinical characteristics and outcomes of MRSA bacteremia patients with different SCCmec types.

Clinical syndrome,n (%)	SCC*mec* II n = 30	SCC*mec* III n = 87	SCC*mec* IV n = 22	SCC*mec* V n = 20	Total n = 159	*P*
Infection focus* ^1^						
Primary bacteremia	6 (20.0)	15 (17.2)	6 (27.3)	5.(25.0)	32 (20.1)	0.695
Skin and soft tissue	0 (0)	6 (6.9)	5 (22.7)	3.(15.0)	14 (8.8)	0.015
Central catheter related infection	13 (43.3)	35 (40.2)	7 (31.8)	3 (15.0)	58 (36.5)	0.152
Surgical site or prosthetic infection	5 (16.7)	12 (13.8)	1 (4.5)	0 (0)	18 (11.3)	0.187
Pneumonia	11 (36.7)	27 (31.0)	3 (13.6)	6 (30)	47 (29.6)	0.326
Deep-seated infection* ^2^	2 (6.7)	3 (3.4)	6 (27.3)	3 (15.0)	14 (8.8)	0.004
Endocarditis	1 (3.3)	2 (2.3)	3 (13.6)	1 (5.0)	7 (4.4)	0.140
Septic arthritis and osteomyelitis	0 (0)	0 (0)	4 (18.2)	1 (5.0)	5 (3.1)	<0.001
Necrotizing pneumonia and empyema	1 (3.3)	2 (2.3)	0 (0)	2 (10)	5 (3.1)	0.256
Outcome						
Persistent bacteremia* ^3^	4 (13.3)	5 (5.7)	1 (4.5)	1 (5.0)	11 (6.9)	0.532
7-day mortality	6 (20.0)	8 (9.2)	0 (0)	0 (0)	14 (8.8)	0.002
30-day mortality	12 (40)	26 (29.9)	4 (18.2)	8 (40.0)	50 (31.4)	0.309

*^1^Some patients had more than one focus of infection.

*^2^Deep-seated infection not related to surgery or prosthesis.

*^3^Persistent bacteremia (>7 days).

### SCC*mec* II/III vs. SCC*mec* IV/V

In subsequent analyses, we grouped patients with SCC*mec* II and SCC*mec* III isolates together for comparison with patients with SCC*mec* IV and SCC*mec* V isolates. In univariate analysis, age, ICU onset, length of stay before index culture, diabetes mellitus, bedridden status, recent surgery, and catheter-related infection were associated with recovery of SCC*mec* II/III isolates (Table 3). Community onset, skin and soft tissue infection, and deep-seated infection (not related to surgery/prosthesis) were risk factors associated with isolates carrying SCC*mec* IV/V (Table 3). Multivariate analysis revealed four independent factors associated with patients infected by MRSA carrying SCC*mec* II/III: ICU onset (OR, 16.82; 95% CI, 3.52–80.15), length of stay before index culture (OR 1.07; 95% CI, 1.03–1.10), diabetes mellitus (OR, 4.51; 95% CI, 1.51–13.42), and bedridden status (OR, 5.90; 95% CI, 1.61–21.61) (Table 3).

**Table 3:** Results of univariate and multivariate logistic regression analyses (SCC mec II/III isolates vs. SCCmec IV/V isolates).

	Univariate OR (95% CI)	Multivariate OR (95% CI)
ICU onset	17.74 (4.10–76.84)	16.82 (3.52–80.15)
Recent surgery	5.71 (1.29–25.24)	
Bedridden	4.06 (1.35–12.23)	5.90 (1.61–21.61)
Diabetes	2.38 (1.01–5.61)	4.51 (1.51–13.42)
Catheter-related infection	2.23 (1.00–4.95)	
Length of hospital say before index culture	1.07 (1.04–1.11)	1.07 (1.03–1.10)
Age	1.03 (1.01–1.04)	
Skin and soft tissue infection	0.23 (0.08–0.71)	
Deep-seated infection	0.16 (0.05–0.52)	
Community-onset	0.15 (0.07–0.33)	

In univariate analysis, MRSA bacteremia patients with SCC*mec* II/III isolates had more catheter-related infections and had more often had recent surgery, and patients with SCC*mec* IV/V isolates had more skin and soft tissue infections and more deep-seated infections. However, these associations failed to reach significance in multivariate analysis.

### Mortality Analysis

**Figure 2:**
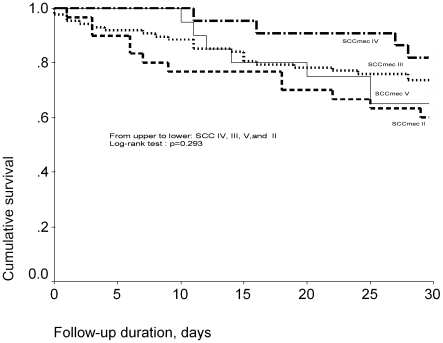
Thirty-day Kaplan-Meier survival curves for MRSA bacteremia patients according to SCCmec type.

**Figure 3:**
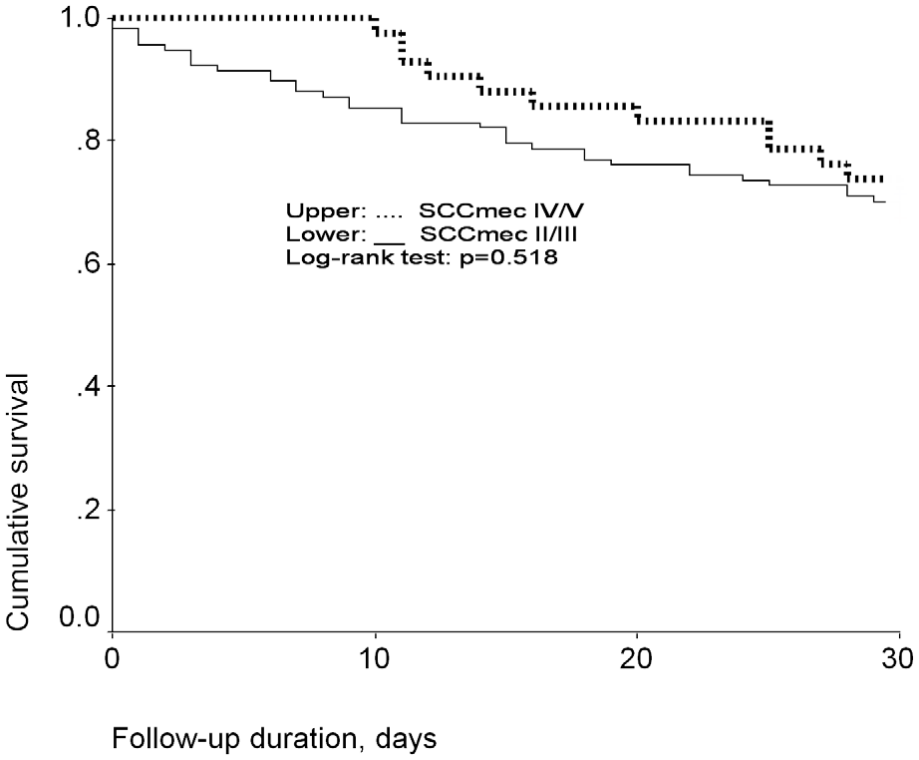
Thirty-day Kaplan-Meier survival curves for MRSA bacteremia patients: SCCmec II/III vs. SCCmec IV/V.

Figure 2 shows the Kaplan-Meier survival curves for MRSA bacteremia patients with isolates carrying different SCC*mec* types. The 30-day cumulative survival was 60%, 70.1%, 81.8%, and 60% for patients with SCC*mec* II, III, IV, and V isolates, respectively. There was no significant difference in survival (*P* = 0.293, log-rank test). The 30-day cumulative survival was 67.5% for patients with SCC*mec* II/III isolates and 71.4% for patients with SCC*mec* IV/V isolates; no significant differences emerged (*P* = 0. 403, log-rank test)(Figure 3).

To determine whether the SCC*mec* type would independently affect 30-day mortality, we performed Cox-regression analysis by controlling for age, sex, and comorbidity (Charlson score). After adjustment for age, sex, and comorbidity (Charlson score), the 30-day mortality of patients with SCC*mec* IV/V isolates was not significantly higher than that of patients with SCC*mec* II/III isolates (adjusted hazard ratio, 0.793; 95% CI, 0.406–1.547; *P* = .496).

### Genotype, Antimicrobial Susceptibility, and Virulence Gene Profile of the MRSA Isolates

**Figure 4:**
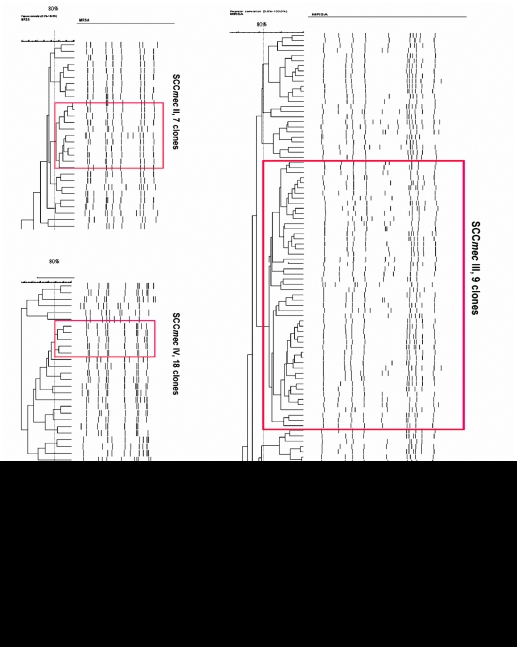
Pulsed-field gel electrophoresis of different SCCmec types; red indicates the major pulsotype.

Table 4 shows the genotype, antibiotic susceptibility, and virulence gene profile of isolates with four different SCC*mec* types. The genotypes of the HA-MRSA strains (SCC*mec* II and III) were more homogeneous (the same identical spa type and pulsotypes) than those of the CA-MRSA strains (SCC*mec* IV and V). Figure 4 shows the PFGE of MRSA isolates containing 4 different SCC*mec* types. The main pulsotypes carried with SCC*mec* II were ST5, spa t002, and agr 2 and were positive for the sec, seg, sei, and tst genes. The main strain carrying SCC*mec* IV and V was ST59 and was positive for spa t437, agr 1, and seb. The major differences in virulence gene profiles and antibiotic susceptibility between SCC*mec* IV and SCC*mec* V isolates were the pvl carrier rate (9% vs. 85%) and gentamicin susceptibility (36% vs. 90%), respectively. The major strain carrying SCC*mec* III was spa ST239 and was positive for spa t037, agr1, and sea. Isolates with SCC*mec* III were often multi-drug resistant except for rifampin.

**Table 4:** Molecular typing, antimicrobial susceptibility, and virulence genes of MRSA isolates with different SCCmec types.

	SCC*mec* II n = 30	SCC*mec* III n = 87	SCC*mec* IV n = 22	SCC*mec* V n = 20
Major *spa* type (%) MLST*	t002 (97%) ST5	t037 (93%) ST239	t437 (68%) ST59	t437 (75%) ST59
Major agr type (%)	agr 2 (93%)	agr 1 (98%)	agr 1 (77%)	agr 1 (85%)
Antibiotic susceptibility profile				
Oxacillin MIC≥128 µg/mL (%)	100	99	0	0
Vancomycin MIC = 2 µg/mL (%)	7	30	0	0
Susceptible rate to non-β-lactam (%)				
Erythromycin	0	0	9	10
Clindamycin	0	18	23	15
Gentamicin	0	1	36	90
Levofloxacin	0	0	100	90
Ciprofloxacin	0	0	100	90
Trimethoprim-sufamethoxazole	100	0	100	100
Minocycline	100	62	91	100
Tetracycline	100	0	41	40
Rifampin	53	83	96	80
Virulence gene				
*pv l* (%)	0	0	9	85
Enterotoxin gene (*sea-sej,tst*)	*sec*: 77% *seg:* 100% *sei:* 100% *tst:* 87%	*sea*: 87% *seb*: 2% *seg*: 1% *sei*: 1%	*seb*: 68% *sec*: 9% *seg*: 23% *sei*: 23%	*seb*: 80% *seg*: 10% *sei*: 10% *sej*: 10%

*We only performed MLST on strains from major pulsotypes for the different SCCmec types.

## Discussion

We conducted a large one-year retrospective study in a medical center in Taiwan that included 159 consecutive adult patients with MRSA bacteremia. We recorded bacterial genotyping results, antibiotic susceptibility, and virulence gene profiling results for each patient. In this study, the percentage of MRSA bacteremia patients with community-onset, healthcare-associated MRSA with SCC*mec* IV/V was 48%; among patients with hospital-onset, healthcare-associated MRSA, the frequency was 17%. This finding is similar to those of previous studies in US populations that found that CA-MRSA strains (USA 300) are an important cause of healthcare-related infections [8]–[10], [29], [30]. We compared the demographic characteristics, risk factors, and outcomes of adult MRSA bacteremia patients from whom strains with different SCC*mec* types were isolated (SCC*mec* II, III, IV, and V). Microbiological study of the isolates showed that the major clones of CA-MRSA strains in Taiwan (SCC*mec* IV/V, spa t437, ST59 with or without the pvl gene) or HA-MRSA strains in Taiwan (SCC*mec* III, spa t037, ST239 and SCC*mec* II, spa t002, and ST5 with the tst gene) differed from those in the United States and Europe. In addition, we found the demographic and clinical characteristics of patients with MRSA bacteremia differed according to specific SCC*mec* subtype.

Two recent studies of MRSA bacteremia used the USA300 PFGE pattern and typical antibiotic phenotype for differentiating CA-MRSA and HA-MRSA strains [8]–[9]. The association we identified between SCC*mec* II/III and ICU onset and length of stay before index culture was similar to results from previous studies [8]–[10], [30]. Previous studies identified a trend between a longer hospital stay before index culture and greater risk of ICU admission in HA-MRSA bacteremia patients [8], [9]. Our cohort also showed longer hospitalization before index culture and more cases of ICU onset. In our study, the number of days the patient was bedridden and diabetes mellitus were other risk factors associated with SCC*mec* II/III isolates. SCC*mec*types IV and V are considerably smaller than SCC*mec* elements I–III. Our finding support the idea that HA-MRSA strains do not survive well in the community setting and that antibiotic selective pressure or cross-transmission in the hospital were needed for the survival of HA-MRSA strains [17].

Little has been published regarding the role or association of specific MRSA genotypes with particular presentations. Fowler et al. showed that MRSA ST30 was associated with a significant trend towards higher levels of hematogenous complications [31]. Edgeworth et al. suggested that the ST239 strain was associated with an increased rate of vascular access device-related bacteremia [32]. In univariate analysis in this study, the bacteremia patients with HA-MRSA strains (t002, ST5 and t037, ST239) had more catheter-related infections, and patients with CA-MRSA strains (mainly spa t437 and ST59) showed a trend towards more skin and soft tissue infections and deep-seated infections; however, neither association reached significance in multivariate analysis. Our findings support previous reports that CA-MRSA strains are highly associated with skin and soft tissue infection, osteomyelitis, and necrotizing pneumonia, although the CA-MRSA strain in Taiwan was not USA300 [1]–[5]. Although our investigation only included patients with MRSA bacteremia, it extended the finding that certain *S. aureus* genotypes are more likely to be associated with some clinical syndromes or with infection severity [31]–[33]. Several studies suggest that CA-MRSA strains harboring the smaller SCC*mec* type IV element grow faster and achieve higher infection burdens than nosocomial MRSA strains [34]–[38]. In agreement with this observation, we found that there were more deep-seated infections involving MRSA with SCC*mec* IV/V than other subtypes. However, in our study MRSA isolates with SCC*mec* IV/V were not more lethal than SCC*mec* II/III isolates. This may be due to more severe comorbidities and more isolates with high vancomycin MIC (MIC = 2) in patients with MRSA carrying SCC*mec* II/III.

Our study had some limitations. First, the SCC*mec* and genotype distribution was different than that reported in other parts of the world, so our results cannot be generalized to populations in which the distribution of CA-MRSA and HA-MRSA strains differs from Taiwan. Second, differences in infection presentation and outcome may be explained by factors other than SCC*mec* genotype, such as virulence genes, antibiotic MIC for the bacterial isolates, adequate infection drainage, and empirical vancomycin therapy. The study size may be too small to address associations between genotype and clinical syndromes or severity.

In conclusion, in this study of 159 adult MRSA bacteremia patients, specific demographic and clinical risk factors were found to predict recovery from bacteremia caused by MRSA with different SCC*mec* types. MRSA isolates carrying SCC*mec* II/III were found more frequently in patients with significant comorbidities and prolonged hospitalization. Community onset, skin and soft tissue infection, and deep-seated infection best predicted MRSA isolates carrying SCC*mec* IV/V.
